# Comparing Digital Therapeutic Intervention with an Intensive Obesity Management Program: Randomized Controlled Trial

**DOI:** 10.3390/nu14102005

**Published:** 2022-05-10

**Authors:** Katarína Moravcová, Martina Karbanová, Maxi Pia Bretschneider, Markéta Sovová, Jaromír Ožana, Eliška Sovová

**Affiliations:** 1 Department of Exercise Medicine and Cardiovascular Rehabilitation, University Hospital Olomouc, I. P. Pavlova 6, 779 00 Olomouc, Czech Republic; marketa.sovova@fnol.cz (M.S.); jaromir.ozana@fnol.cz (J.O.); eliska.sovova@fnol.cz (E.S.); 2 Department of Health, Faculty of Medicine, Masaryk University, Kamenice 5, 625 00 Brno, Czech Republic; martina.karbanova@med.muni.cz; 3 First Faculty of Medicine, Charles University in Prague, Kateřinská 32, 121 08 Prague, Czech Republic; 4 Department for Prevention and Care of Diabetes, Department of Medicine III, Faculty of Medicine Carl Gustav Carus, Technische Universität Dresden, Fetscherstrasse 74, 01307 Dresden, Germany; maxi.bretschneider@mailbox.tu-dresden.de

**Keywords:** obesity, diabetes mellitus type 2, prevention, digital therapeutics, mobile application, randomized controlled trial, lifestyle intervention, metabolic syndrome, insulin resistance

## Abstract

In this study, we evaluated whether the digital program Vitadio achieves comparable results to those of an intensive in-person lifestyle intervention in obesity management. This is a 12-month prospective, randomized controlled trial. Obese patients with insulin resistance, prediabetes or type 2 diabetes were included. The intervention group (IG) used Vitadio. The control group (CG) received a series of in-person consultations. Body weight and various metabolic parameters were observed and analyzed with ANOVA. The trial is ongoing and the presented findings are preliminary. Among 100 participants (29% men; mean age, 43 years; mean BMI, 40.1 kg/m^2^), 78 completed 3-month follow-up, and 51 have completed the 6-month follow-up so far. Participants significantly (*p* < 0.01) reduced body weight at 3 months (IG: −5.9 ± 5.0%; CG: −4.2 ± 5.0%) and 6 months (IG: −6.6±6.1%; CG: −7.1 ± 7.1%), and the difference between groups was not significant. The IG achieved favorable change in body composition; significant improvement in TAG (−0.6 ± 0.9 mmol/l, *p* < 0.01), HDL (0.1 ± 0.1%, *p* < 0.05), HbA1c (−0.2 ± 0.5%, *p* < 0.05) and FG (−0.5 ± 1.5 mmol/l, *p* < 0.05); and a superior (*p* = 0.02) HOMA-IR reduction (−2.5 ± 5.2, *p* < 0.01). The digital intervention achieved comparable results to those of the intensive obesity management program. The results suggest that Vitadio is an effective tool for supporting patients in obesity management and diabetes prevention.

## 1. Introduction

Obesity is a chronic disease related to numerous other conditions, including type 2 diabetes, cardiovascular disease and cancer [1,2]. Addressing obesity together with a cluster of cardiometabolic risk factors commonly referred to as metabolic syndrome is an essential part of prevention and management of many chronic diseases [3,4]. There is particularly strong evidence that obesity management can delay the onset of type 2 diabetes and lead to improved diabetes control, as well as a reduction in cardiovascular risk factors and medication use in patients with diagnosed type 2 diabetes [5,6,7].

Obesity has multifactorial etiology, including genetics, metabolism and behavioral elements. Therefore, a multimodal lifestyle intervention covering diet, exercise and behavioral therapy is recommended as a part of obesity treatment [8,9]. According to numerous studies, lifestyle intervention promotes increased weight loss in obese patients compared to standard care [10]. In some trials, the intervention groups also improved glycemic control, lipids profile, blood pressure and quality of life more than the control groups [11,12]. Despite considerable evidence on the clinical effectiveness of lifestyle interventions, many barriers hinder effective implementation of such interventions in routine clinical practice. Healthcare providers perceive a lack of appropriate training, confusion about congruence with their role and a lack of confidence in opening lifestyle conversations with patients. Patients on the other side struggle with physiological mechanisms aggravating lifestyle change, as well as various psychosocial challenges between physician visits, such as self-regulation for behavior change, competence beliefs, ability to change or interference with social relationships [13,14,15].

Digital health applications have the potential to provide the missing link between professional care and the individual patient. Applications can deliver lifestyle interventions digitally without burdening physicians and can provide patients with ongoing support in their home environment. They are able to encourage patients to adopt healthier behaviors and increase physical activity [16,17,18]. Some principles and design aspects, such as structured guidance, ongoing feedback or access to human support, have been shown to be associated with positive clinical outcomes [19]. However, freely available health applications vary in quality and lack the trust of healthcare professionals. Evidence on the safety and effectiveness of digital health applications remains limited. This gap must be addressed to enable implementation of digital health in daily practice [20,21].

Vitadio is an evidence-based digital care program empowering patients in effective self-management and lifestyle change. The application is based on a multimodal therapy approach and offers a combination of interactive education, tracking tools and communication functions. It supports patients to adopt a healthy routine by employing strategies such as gamification, feedback, personalized goal setting and social interactions. In this study, we aimed to evaluate whether Vitadio achieves comparable results in obesity management to those of an intensive in-person lifestyle modification program.

## 2. Materials and Methods

### 2.1. Study Design

The study design is a prospective, double-armed, randomized controlled trial with an active control group. The primary objective is to evaluate whether the effect of using Vitadio is comparable to that of participating in an intensive individualized weight reduction program administered at a specialized clinic in-person. The trial is ongoing, is registered with ClinicalTrials.gov, accessed on 1 March 2022, (NCT04573296) and was approved by the Ethics Committee of the University Hospital Olomouc (ref. number 10/20) in January 2020. 

### 2.2. Participants 

#### 2.2.1. Eligibility

Obese patients (defined as BMI over 30 kg/m^2^) older than 18 years were included in the study. To evaluate the effect of the intervention on both progression and prevention of type 2 diabetes, several stages of type 2 diabetes were included. Only patients with one of the following conditions were included: diagnosed type 2 diabetes mellitus or prediabetes (defined as fasting glucose within the range of 5.6–6.9 mmol/L or oral glucose tolerance test (OGTT) within the range of 7.8–11.0 mmol/L) or insulin resistance (IR) (defined as HOMA-IR > 2.7) [22]. All participants owned a smartphone compatible with Vitadio application and spoke fluently Czech. 

To ensure the integrity of the study, the following exclusion criteria were applied: insulin therapy, severe liver or kidney disease, age older than 60 years, pregnancy, steroid therapy, and inability or unwillingness to use Vitadio or to comply with study procedures. 

#### 2.2.2. Recruitment

The participants were recruited at the Department of Exercise Medicine and Cardiovascular Rehabilitation. Patients were screened during routine clinical practice, and those who met the eligibility criteria were offered participation in the study. Those who signed the informed consent joined the study. Participants were numbered according to the order of admission and randomly assigned in a 1:1 ratio to the intervention group (IG) or the control group (CG). Blinding of physicians and participants was not feasible due to the nature of the intervention.

### 2.3. Study Procedures

After treatment assignment, a baseline visit was conducted, which included collection of demographic and anamnestic data. The study procedure includes 4 visits: at baseline, after 3 months, after 6 months and after 12 months, with anthropometric and laboratory examinations performed during each visit. Blood specimens were obtained after a 12–14 h fast. Glucose was measured by a glucose hexokinase method [23]. Lipids were analyzed using an enzymatic colorimetric test [24]. Serum insulin was measured by a two-step sandwich enzyme immunoassay using monoclonal antibodies. HOMA-IR was calculated using the formula of Matthews et al. [25]. Body composition was measured by bioelectrical impedance analysis using InBody 370 with 15 impedance measurements at 5 body segments and a tetrapolar 8-point tactile electrode system.

Successful study completion is defined as the point at which the participant has completed all phases of the study, including the final visit at 12 months. Participants did not receive any financial or non-financial reward. Participants withdrawing their consent or participants withdrawn by the investigator did not proceed to the final visit.

### 2.4. Intervention

The intervention group received the Vitadio app without any in-person lifestyle consultation. Vitadio is a certified class I medical device employing a multimodal therapy approach to provide individualized support in lifestyle modification and self-management (see Appendix A). The digital care program consists of a 3-month intensive phase followed by a 3-month sustaining phase. Participants were able use the app for 12 months to maintain access to their logged data. The application guides patients through the program using a system of daily tasks and automated messages. The tasks develop according to the patient’s choices and progress in the program. Tasks focus on establishing a healthy routine, and their completion is positively reinforced by gamification principles. Patients follow an interactive educational course covering topics including motivation, healthy eating patterns, physical activity, sleep hygiene, mental wellbeing and social aspects of life with diabetes. The lessons are implemented using gamified personal goals that help patients root important habits into their daily life. Patients are also nudged to monitor their physiological and lifestyle parameters. The program is enhanced by a set of human support features. To ensure patient safety and enhance effective use of the program, a qualified personal dietitian was available on chat to answer patient questions. Optionally, participants were able to book an onboarding phone consultation with their dietitian. To improve adherence, patients can join a peer support group to share encouragement and experience.

### 2.5. Comparator

The control group received 5 in-person lifestyle consultations over the course of 6 months. In the subsequent 6 months, participants were able to reach out to their educator for additional support in sustaining their lifestyle. The education was provided by a physician, dietitian and/or educational nurse at the Department of Exercise Medicine and Cardiovascular Rehabilitation. The recommendations followed principles of healthy eating patterns, such as the dietary approach to stop hypertension (DASH) or a Mediterranean diet (MED). Restrictive weight-loss diets or meal replacements were not included. Additionally, participants received an online diary tool for recording meals, which included macronutrients composition analysis, energy intake calculation, recipes and sample diet plans. They had an option to ask the educator for additional information and feedback on their diet using this online tool.

### 2.6. Outcome Measurement

With this study, we aimed to evaluate whether Vitadio achieves superior results to those achieved with a structured, in-person lifestyle modification program focused on weight reduction. Thus, the primary outcome is to analyze the effect of Vitadio on weight reduction when compared to an in-person program. 

The secondary outcomes include the impact on body composition, waist circumference, glucose and lipid metabolism parameters and liver function parameters. Physical fitness (assessed by spiroergometry) and sleep apnea were measured in the study but are not presented in this paper.

To assess user acceptance of Vitadio, user data, such as retention and drop-out rates, frequencies of interactions and compliance with the program (such as reading lessons, achieving personal goals, compliance with self-monitoring, etc.), were analyzed.

### 2.7. Sample Size

The null hypothesis is that Vitadio is less effective than (inferior to) the structured in-person obesity management program. The alternative hypothesis is that the effect of Vitadio on weight loss is at least as effective as the compared treatment, i.e., not worse than by the prespecified non-inferiority margin. There are no clear guidelines for selecting the margin. However, the margin must not be higher than the entire effect size and also should be lower than what is considered clinically meaningful in a disease area [26]. For these reasons, 3% weight loss was chosen as the margin, which is considered minimal weight reduction associated with clinical benefits [11]. Both groups are hypothesized to lose 5–9% of their body weight over the 6 months with a standard deviation of 5% [27]. Assuming equivalent weight loss in both groups, 35 participants are required in each group (70 in total) to demonstrate non-inferiority, given a 3% margin, 80% power and 5% significance level. We assumed a 40% attrition rate based on the metanalysis of 17 studies showing average attrition of 43% for app-based interventions [28]. Therefore, 100 participants were recruited.

### 2.8. Statistical Analysis

A summary of baseline data (demographic and anamnestic data and laboratory parameters) was developed. Continuous variables were described by standard descriptive statistics containing mean and standard deviation. The normal distribution in continuous variables was tested by the Shapiro–Wilk test. Categorical variables were described according to the frequency of occurrence (absolute numbers and percentages) using the chi-square test. For descriptive statistics, a 95% confidence interval was used to assess a significant difference between groups.

Complete case analyses for 3- and 6-month data were presented separately to prevent data loss due to significant attrition between 3- and 6-month follow-ups. Only complete cases are reported for all analyses, and no imputation method was used. A two-way repeated measures ANOVA with Bonferroni correction of the post hoc tests was used to assess the overall significance of differences in metabolic parameters. Changes in other parameters were evaluated using paired t-tests or Wilcoxon signed-rank tests as appropriate. An alpha of 0.05 was used as the cut-off for statistical significance. All analyses were performed using R-Software version 4.0.3 (developed by R Foundation for Statistical Computing, Vienna, Austria).

## 3. Results

### 3.1. Participant Characteristics

The trial is ongoing. So far, 100 participants have been recruited for the study: 50 for the intervention group and 50 for the control group. The 3-month and 6-month follow-up visits were completed by 40 and 28 participants in the intervention group and 38 and 23 participants in the control group, respectively. At the time of the analysis, the 6-month laboratory examination was available only for a fraction of participants (IG: 18, CG: 9); thus, only the 3-month laboratory measurement is evaluated in this paper. The 6-month attrition rate was higher in the control group (IG: 36%, CG: 45%). The most frequent reasons for dropout were associated with the COVID-19 pandemic (Table 1).

The sample contains significantly more women (71%), and the average age is 43.3 ± 9.5 years. A majority of participants have a high school education (75 participants). The study population has an average BMI of 40.1 kg/m^2^ and generally shows components of metabolic syndrome, such as central obesity (average waist circumference of 117 cm), insulin resistance (average HOMA-IR of 5.6) or raised triacylglycerols (average TAG of 2.1 mmol/L), as well as low concentrations of HDL cholesterol (the average HDL of 1.1 mmol/L is considered low, given the significant overhang of women in the sample) [3,29]. A majority of participants suffer from insulin resistance (67 participants), as compared to prediabetes (23 participants) and type 2 diabetes (10 participants). Participants with diabetes are mostly treated with metformin, and no participant with prediabetes uses diabetes medication. There was not any significant difference between the intervention and control groups across any of the observed parameters. Table 2 shows a summary of the baseline characteristics of the entire sample.

### 3.2. Effects on Body Weight

Both groups significantly reduced their body weight based on both 3- and 6-month complete case analyses. A repeated measures ANOVA showed a significant time effect (F(1.27,62.3) = 52.34, *p* < 0.001, *η*^2^ = 0.52) but no group–time interaction or group effects (*p* > 0.05). Both groups achieved the largest weight reduction in the first 3 months, followed by another smaller reduction between the 3rd and 6th months (Table 3).

Interestingly, the 3-month weight reduction significantly differed based on the subsample of the analysis (3- or 6-month complete cases) (Table 3 and Table 4). The intervention group achieved a 3-month weight reduction of −6.5 ± 4.3 kg (−5.5% of bodyweight) based on the 6-month subsample and −7.1 ± 6.5 kg (−5.9% of bodyweight) based on the 3-month subsample, whereas the control group achieved a 3-month weight reduction of −6.0 ± 6.3 kg (−5.1% of bodyweight) based on the 6-month subsample and −4.8 ± 5.8 kg (−4.1% of bodyweight) based on the 3-month subsample. The difference is driven by a differing nature of attrition. In the intervention group, dropping-out participants achieved higher weight reduction (−8.7 kg or −6.5% of bodyweight), whereas in the control group, the dropping-out participants achieved minor weight loss (−1.7 kg or −1.5% of bodyweight). Consequently, the 6-month reduction was higher in the control group (IG: −6.6 ± 6.1%, CG: −7.1 ± 7.1%). Any difference between groups was not significant.

The average BMI decreased by −2.7 ± 2.2 kg/m^2^ (*p* < 0.001) in the intervention group and −2.9 ± 3 kg/m^2^ (*p* < 0.001) in the control group over the 6 months. In total, 18% of the participants in the intervention group and 4% of the participants in the control group reduced their BMI below the obesity threshold (BMI under 30 kg/m^2^). 

The margin for evaluating whether the digital intervention is at least as effective as the in-person intervention was specified as a 3% body weight reduction (see Section 2.7). Given this margin, Vitadio achieved non-inferior results compared to the intensive in-person lifestyle modification program administered at a specialized clinic at both 3- and 6-month follow-ups (*p* < 0.001).

### 3.3. Effects on Anthropometabolic Parameters

Participants in both groups reduced their waist circumference at 3 months (IG: −5.6 ± 6.5 cm, *p* < 0.01; CG: −4.1 ± 4.3 cm, *p* < 0.01) and 6 months (IG: −6.3 ± 5.9 cm, *p* < 0.001; CG: −7.0 ± 5.4 cm, *p* < 0.001). The inconsistency between 3-month (Table 4) and 6-month (Table 3) complete case analyses is again driven by the different success rates of dropping-out participants. Change in body composition was measured by change in muscle mass and body fat. The intervention group retained muscle mass (+0.05 ± 2.5 kg, *p* = 0.92) while reducing body fat (−7.5 ± 7.0 kg, *p* < 0.001), whereas the control group reduced both muscle mass (−0.4 ± 1.4 kg, *p* = 0.17) and body fat (−7.4 ± 7.0 kg, *p* < 0.001). When looking at 3-month complete case analysis, the intervention group achieved significantly (*p* = 0.02) superior body fat reduction compared to the control group (IG: −6.7 ± 5.2 kg, *p* < 0.01; CG: −4.1 ± 4.5 kg, *p* < 0.01).

### 3.4. Effects on Parameters of Glucose Metabolism

Parameters of glucose metabolism were analyzed to evaluate the effect of Vitadio on the progression of type 2 diabetes. The intervention group achieved significant improvement across all glycemic parameters: HOMA-IR change: −2.5 ± 5.2 (*p* < 0.01); HbA1c change: −0.2±0.5% (*p* < 0.05); and fasting glucose change: −0.5 ± 1.5 mmol/L (*p* < 0.05). The control group achieved a significant reduction in HbA1c only (−0.2 ± 0.4%, *p* < 0.05). The change in HOMA-IR achieved by the intervention group was superior to that of the control group (*p* = 0.02). Despite a reduction in body weight, a statistically non-significant increase in HOMA-IR (1 ± 5.7, *p* > 0.05) can be observed in the control group. The distribution of HOMA-IR change differs between the intervention and control groups. Nearly all intervention participants (29 out of 33) have shown either a decrease or a marginal increase in HOMA-IR (0.3 or lower). No outliers were observed in this group in either direction. In the control group, fewer participants achieved lower or stable HOMA-IR (15 out of 25). Several participants have shown a significant increase in the index—some of them, very substantial (4 participants by 4.7 or more). The results are summarized and presented according to disease stage (non-diabetics and diabetics) in Table 5. Due to the low number of participants with type 2 diabetes, the results of subanalysis of this group are not statistically significant and cannot be reliably interpreted.

### 3.5. Effects on Lipid Parameters

The intervention group achieved a significant improvement in triacylglycerols (−0.6 ± 0.9 mmol/L, *p* < 0.01) and HDL cholesterol (0.1 ± 0.1 mmol/L, *p* < 0.05). The change in total cholesterol and LDL cholesterol was not statistically significant. The control group did not achieve a significant change in lipid profile (Table 6).

### 3.6. Effects on Liver Enzymes

Both groups achieved a modest reduction in liver enzymes. Participants reduced both alanine transaminase (ALT) (IG: −0.1 ± 0.4 µkat/L, *p* > 0.05; CG: −0.1 ± 0.2 µkat/L, *p* < 0.05) and aspartate transaminase (AST) levels (IG: −0.1 ± 0.4 µkat/L, *p* < 0.05; CG: −0.1 ± 0.2 µkat/L, *p* > 0.05). Both groups achieved the same decrease (−0.1 ± 0.2 µkat/L) in gamma-glutamyltransferase (GGT) level, but this reduction was not significant (Table 6).

### 3.7. Adherence to Digital Therapy

The app-generated data were analyzed for all participants who had started using Vitadio, regardless of attrition. An interaction with the program was defined as at least two actions within the app to exclude accidental opening of the app with no further user activity. Out of 46 participants in the intervention group, 37% of participants used the app daily (measured as at least one interaction every day), and 83% of participants used the app on an almost daily basis (measured as at least one daily interaction for at least 80% of days).

Participants actively used all the main features of the program: 89% of participants completed all educational materials; 93% of participants actively set their own goals (measured as least five goals over the 3-month program); and 85%, 69% and 38% of participants used the app to monitor their body weight, waist circumference and glycaemia, respectively, at least biweekly. More than 98% participants monitored their mood using an emoji system, resulting in an average mood of 3.3 out of 5 (where 5 indicates the best mood).

Participants also actively used a meal photo diary, resulting in an average of 301 meal photos per participant in 3 months. Self-evaluation of eating habits increased from 6.6/10 during the first month to 6.9/10 during the last month of the 3-month observation period. More than 70% of participants used step tracking via synchronization with the Google Fit/Apple Health application. The average number of recorded daily steps was 5594 over the first 3 months.

## 4. Discussion

In this study, we compared the digitally administered lifestyle intervention with a structured in-person obesity management program. We measured the impact of both interventions on body weight, body composition, glucose metabolism and other metabolic parameters. The principal findings are that the digital app-based therapy, Vitadio, achieves non-inferior results compared to those of lifestyle intervention (*p* < 0.001). In agreement with the findings of Spring et al., our results suggest that digital therapies do not deliver better results than intensive in-person counseling but might be superior in terms of cost effectiveness and provide better clinical results than standard non-intensive treatment [30].

Participants in both groups significantly (*p* < 0.01) reduced their body weight at 3-month and 6-month follow-up. Furthermore, both groups significantly reduced the BMI index at 6 months, although more participants in the intervention group (IG: 18% vs. CG: 4%) reduced their BMI below the obesity threshold (BMI under 30 kg/m^2^). Such weight loss is associated with many clinical benefits, including glycemic improvement, reduction in blood pressure and increased quality of life, even for patients with higher BMI levels (>40 kg/m^2^). Moderate weight loss (5–10%) has been shown to be associated with reduced healthcare costs [31]. Further research is needed to investigate the effects of digital lifestyle interventions in comparison with standard of care and on individuals with lower BMI levels. 

The 6-month attrition rate was higher in the control group (IG: 36%, CG: 45%), and more than 25% of dropouts were associated with the coronavirus pandemic. The nature of attrition between 3- and 6-month follow-up differs between the groups. In the intervention group, dropping-out participants achieved a higher weight reduction (−6.5% of bodyweight), whereas in the control group, the dropping-out participants achieved minor weight loss (−1.5% of bodyweight). Consequently, the 3-month measurement differs based on the subsample (3- or 6-month complete cases). More attention should be paid to the attrition rate in further research.

Favorable changes in body composition, i.e., losing fat mass while maintaining lean body mass, are important aspects of healthy weight loss. Protection of lean body mass is crucial for sustainability of weight reduction and offsetting of potential negative health consequences [32,33]. The intervention group retained muscle mass while reducing body fat, whereas the control group reduced both muscle mass and body fat. Considering the 3-month complete case analysis, the intervention group achieved significantly (*p* = 0.02) superior body fat reduction compared to the control group. 

Abdominal obesity is a key factor associated with each of the other metabolic syndrome components. Its reduction is an essential part of metabolic syndrome treatment, and it is associated with improvements in cardiometabolic risk factors [34]. Both groups reduced their waist circumference at 3 months and 6 months. Digital intervention had a significant positive effect on triacylglycerol levels and HDL cholesterol, which are other typical components of metabolic syndrome [3,29]. The control group did not achieve any significant change in lipid profile. In line with previous findings of St George et al. [35], both groups also achieved a modest reduction in liver enzymes (ALT and AST).

The intervention group achieved a significant reduction across all glycemic parameters: HOMA-IR: −2.5 ± 5.2 (*p* < 0.01); HbA1c: −0.2 ± 0.5% (*p* < 0.05); and fasting glucose: −0.5 ± 1.5 mmol/L (*p* < 0.05). The control group achieved a significant reduction in HbA1c only (−0.2 ± 0.4%, *p* < 0.05). Given the majority of participants in the sample are non-diabetic, HOMA-IR was selected as the most relevant parameter for evaluation of the intervention effect on glucose metabolism. High HOMA-IR values are associated not only with poor glycemic control but also with metabolic syndrome [36]. The intervention group achieved a superior change in HOMA-IR compared to the control group (*p* = 0.02). Particular attention was paid to an unexpected increase in HOMA-IR despite the reduction in body weight in the control group. Sensitivity analysis was performed by excluding one participant in the control group with an increase outside the range of ±3 standard deviations from the mean. The results of the analysis were robust to this outlier, the increase in HOMA-IR was still observed and the difference between the groups remained significant. One possible explanation is that the intervention group achieved healthier weight reduction characterized by reducing body fat while maintaining lean body mass. Favorable changes in body composition and a greater (non-significant) decrease in visceral fat measured by waist circumference support this hypothesis. Muscle is important in the pathophysiology of insulin resistance; thus, by failing to protect muscle mass, participants in the control group might have a disadvantage in terms of insulin resistance. This phenomenon should be analyzed in detail in future research. The results indicate that Vitadio is effective in lowering insulin resistance and might have potential to become a tool for diabetes prevention.

The adherence of the participants to the digital program was high; 83% of participants used the app on an almost daily basis, and 37% participants used the app daily. The share of daily users is comparable to daily engagement with Twitter [37]. Participants engaged with all the main features of the program and used the app to monitor their health status and lifestyle data. The high adherence to the app demonstrates the ability of digital tools to provide ongoing support in lifestyle change.

### Strengths and Limitations

The main strength of this study is that it was designed as a randomized controlled trial. Both intervention and control groups are balanced in terms of sex and age, as well as wide range of metabolic parameters. We observed a complex set of anthropometric and laboratory parameters to perform complex evaluation of effects of the intervention. The study is still ongoing, with a relatively long observation period (12 months), although for the present analysis, only 6-month data were available. Additionally, the data from the app were analyzed to assess participants’ adherence.

The trial is ongoing; thus, the presented data are preliminary. The risk of selection bias, single-center design and relatively high baseline BMI (40 ± 6.2 kg/m^2^) might limit the generalizability of the results. However, for individuals with higher BMI, the ability to lose the same proportion of weight with lifestyle intervention is equal to that of those with lower BMI levels, and there is equal benefit in terms of risk-factor improvement with modest weight loss [31]. Due to the nature of the intervention, participants and clinicians were not blinded to assessment. Moreover, the control group received intensive in-person counseling instead of standard care; thus, achieving superiority cannot be anticipated [30]. In terms of glycemic control, the small number of subjects with type 2 diabetes is also the main limitation. In combination with large baseline differences between the intervention and the control group (especially in HOMA-IR), the reliability of the results in the diabetic population remains limited. Because the majority of patients are diagnosed with insulin resistance or prediabetes, the study demonstrates the effect of obesity management in diabetes prevention than treatment. In the scope of this analysis, we do not have a sufficient amount of 6-month laboratory data; thus, only the 3-month laboratory data were evaluated.

## 5. Conclusions

The study results demonstrate that the digital lifestyle intervention is as effective in reducing body weight and improving various metabolic parameters as the intensive in-person obesity management program. Moreover, the findings indicate that Vitadio digital therapy achieves superior results in decreasing insulin resistance compared to in-person intervention. Vitadio was well-accepted by patients and has potential to increase accessibility of lifestyle intervention. More research is needed to analyze the target patients and investigate the effects of digital lifestyle interventions in comparison with standard of care. The study is ongoing, and the presented results are preliminary.

## Figures and Tables

**Table 1:** Participant randomization, follow-up and attrition.

Randomized (*n* = 100)	
Treatment (*n* = 50)	Control (*n* = 50)
3-month follow-up	
Attrition (*n* = 6) ○ Health reasons (*n* = 3) ○ Pregnancy (*n* = 1) ○ Work reasons (*n* = 1) ○ Lost to follow-up (*n* = 1) Have not reached 3 months yet (*n* = 4) Analysed (*n* = 40)	Attrition (*n* = 8) ○ Health reasons (*n* = 4) ○ Other reasons (*n* = 2) ○ Lost to follow-up (*n* = 2) Have not reached 3 months yet (*n* = 4) Analysed (*n* = 38)
6-month follow-up	
Attrition * (*n* = 10) ○ Health reasons (*n* = 2) ○ Pregnancy (*n* = 1) ○ Work reasons (*n* = 3) ○ Other reasons (*n* = 3) ○ Lost to follow-up (*n* = 1) Have not reached 6 months yet (*n* = 2) Analysed (*n* = 28)	Attrition * (*n* = 11) ○ Health reasons (*n* = 2) ○ Work reasons (*n* = 2) ○ Other reasons (*n* = 3) ○ Unspecified reasons (*n* = 4) Have not reached 6 months yet (*n* = 4) Analysed (*n* = 23)

* The dropout analyzed at 6-month follow-up is in addition to the dropout in the first 3 months (e.g., IG cumulative attrition at 6 months is 16 participants, and 6 participants have not reached the 6-month follow-up visit yet).

**Table 2:** Baseline demographic and clinical characteristics.

Characteristics	Overall	Intervention Group	Control Group	*p*-Value between Groups
*n*	100	50	50	
Men (%)	29%	32%	26%	0.66
Age (years)	43.3 ± 9.5	43.3 ± 10.5	43.3 ± 8.4	0.99
Education				
Primary	5	3	2	0.12
High school	75	33	42	
College	20	14	6	
Diabetes progression				
Type 2 diabetes	10	5	5	0.24
Prediabetes	23	15	8	
Insulin resistance	67	30	37	
Diabetes pharmacotherapy (Type 2 diabetes and Prediabetes participants only)				
None	23	15	8	
Metformin	7	3	4	
Sulfonylureas	1	1	0	0.6
Other	2	1	1	
Metabolic parameters				
Body weight (kg)	117.6 ± 20.9	117.5 ± 21.0	117.8 ± 21.0	0.94
BMI (kg/m^2^)	40.1 ± 6.1	40.5 ± 7.1	39.7 ± 5.1	0.51
Waist circumference (cm)	116.8 ± 14.7	118.1 ± 15.4	115.4 ± 14.0	0.36
Muscle mass (kg)	35.8 ± 7.5	35.8 ± 7.3	35.8 ± 7.9	1
Body fat (kg)	53.6 ± 13.4	53.0 ± 15.3	54.3 ± 11.3	0.62
Total cholesterol (mmol/L)	4.9 ± 0.8	4.8 ± 0.9	4.9 ± 0.7	0.74
TAG (mmol/L)	2.1 ± 1.2	2.1 ± 0.9	2.0 ± 1.5	0.70
HDL (mmol/L)	1.1 ± 0.2	1.1 ± 0.2	1.2 ± 0.2	0.21
LDL (mmol/L)	2.8 ± 0.7	2.8 ± 0.8	2.9 ± 0.7	0.58
FG (mmol/L)	5.7 ± 1.3	5.9 ± 1.5	5.6 ± 1.1	0.30
HbA1c (%)	5.6 ± 0.7	5.6 ± 0.7	5.5 ± 0.7	0.52
HOMA-IR	5.6 ± 4.0	6.3 ± 4.8	4.9 ± 2.8	0.06
ALT (µkat/L)	0.8 ± 0.5	0.8 ± 0.6	0.7 ± 0.4	0.36
AST (µkat/L)	0.5 ± 0.3	0.5 ± 0.4	0.5 ± 0.3	0.77
GGT (µkat/L)	0.7 ± 0.6	0.7 ± 0.4	0.7 ± 0.8	0.57

**Table 3:** Descriptive and inferential statistics of the 6-month complete cases (repeated measures ANOVA).

	Intervention Group (*n* = 28)			Control Group (*n* = 23)						Repeated Measures ANOVA
	Baseline	3 Months	6 Months	Baseline	3 Months	6 Months		F	*p*	Partial *η*^2^
Weight (kg)	114.3 ± 20.0	107.8 ± 19.0 *	106.6 ± 19.8 ^	117.6 ± 22.3	111.6 ± 23.2 *	109.3 ± 23.8 *^,^^	Interaction	0.22	0.70	0.00
							Group	0.30	0.59	0.01
							Time	52.34	<0.001	0.52
Waist circumference (cm)	117.6 ± 16.3	112.6 ± 15.3 *	111.3 ± 16.4 ^	114.4 ± 16.6	109.5 ± 16.3 *	107.5 ± 16.4 *^,^^	Interaction	0.19	0.76	0.00
							Group	0.56	0.46	0.01
							Time	57.95	<0.001	0.54
Muscle mass (kg)	35.4 ± 6.9	35.7 ± 6.9	35.5 ± 7.1	36.1 ± 8.4	35.7 ± 8.4	35.6 ± 8.6	Interaction	0.88	0.38	0.02
							Group	0.01	0.91	0.00
							Time	0.32	0.64	0.01
Body fat (kg)	50.8 ± 15.6	44.5 ± 16.2 *	43.3 ± 17.6 ^	53.7 ± 11.1	48.6 ± 11.1 *	46.2 ± 11.8 *^,^^	Interaction	0.43	0.57	0.01
							Group	0.69	0.41	0.01
							Time	54.2	<0.001	0.53

All data are presented as mean ± SD; * significantly different from baseline (*p* < 0.05); ^ significantly different from 3-month follow-up (*p* < 0.05)

**Table 4:** Change in anthropometric parameters in 3-month complete cases.

Parameter	Intervention Group (*n* = 40)			Control Group (*n* = 38)			*p*-Value between Groups
	Baseline Values	3-Month Follow-Up	Change	Baseline Values	3-Month Follow-Up	Change	
Body weight (kg)	119.5 ± 21.4	112.4 ± 20.7	−7.1 ± 6.5 **	116.3 ± 20.7	111.5 ± 21.1	−4.8 ± 5.8 **	0.10
Body weight (%)	-	-	−5.9 ± 5.0 **	-	-	−4.2 ± 5.0 **	0.12
BMI (kg/m^2^)	41.1 ± 7.5	38.9 ± 7.2	−2.2 ± 2.2 **	39.3 ± 4.9	37.7 ± 5.0	−1.6 ± 2.1 **	0.21
Waist circumference (cm)	119.1 ± 16.5	113.5 ± 14.9	−5.6 ± 6.5 **	114.7 ± 14.7	110.7 ± 14.8	−4.1 ± 4.3 **	0.21
Muscles (kg)	36.1 ± 7.0	36.2 ± 6.9	+0.1 ± 2.2	35.7 ± 7.8	35.3 ± 7.7	−0.3 ± 1.4	0.25
Fat (kg)	54.2 ± 16.4	47.5 ± 16.7	−6.7 ± 5.2 **	53.1 ± 10.6	49.0 ± 10.9	−4.1 ± 4.5 **	0.02 *

All data are presented as mean ± SD; * *p* < 0.05; ** *p* < 0.01.

**Table 5:** Change in parameters of glucose metabolism in 3-month complete cases.

Parameter	Intervention Group (*n* = 33)			Control Group (*n* = 25)			*p*-Value between Groups
	Baseline Values	3-Month Follow-Up	Change	Baseline Values	3-Month Follow-Up	Change	
Overall	**n* =* 33			**n* =* 25			
HOMA-IR	6.5 ± 5.3	4.0 ± 2.7	−2.5 ± 5.2 **	4.6 ± 1.9	5.6 ± 5.9	+1.0 ± 5.7	0.02 *
HbA1c (%)	5.7 ± 0.8	5.5 ± 0.4	−0.2 ± 0.5 *	5.6 ± 0.9	5.4 ± 0.7	−0.2 ± 0.4 *	0.88
FG (mmol/L)	6.0 ± 1.8	5.5 ± 0.9	−0.5 ± 1.5 *	5.7 ± 1.5	5.7 ± 1.5	−0.0 ± 0.6	0.09
Nondiabetics	**n* =* 29			*n=* 21			
HOMA-IR	5.6 ± 3.8	4.1 ± 2.8	−1.6 ± 3.4 *	4.6 ± 1.9	5.3 ± 6.2	+0.8 ± 5.9	0.11
HbA1c (%)	5.5 ± 0.4	5.4 ± 0.3	−0.1 ± 0.2	5.4 ± 0.3	5.3 ± 0.2	−0.1 ± 0.3	0.61
FG (mmol/L)	5.6 ± 0.7	5.3 ± 0.5	−0.3 ± 0.5 **	5.3 ± 0.5	5.3 ± 0.6	−0.0 ± 0.6	0.12
Type 2 diabetes	**n* =* 4			**n* =* 4			
HOMA-IR	12.6 ± 10.3	3.5 ± 2.2	−9.1 ± 10.5	4.8 ± 2.1	6.8 ± 4.5	+2.0 ± 5.1	0.13
HbA1c (%)	7.2 ± 1.3	6.2 ± 0.6	−1 ± 1.3	6.7 ± 1.9	6.3 ± 1.4	−0.5 ± 0.7	0.61
FG (mmol/L)	9.3 ± 3.7	7.1 ± 1.1	−2.2 ± 4.0	7.9 ± 2.8	7.8 ± 2.8	−0.1 ± 0.6	0.12

All data are presented as mean ± SD; * *p* < 0.05; ** *p* < 0.01.

**Table 6:** Change in lipid parameters and liver enzymes in 3-month complete cases.

Parameter	Intervention Group (*n* = 33)			Control Group (*n* = 25)			*p*-Value between Groups
	Baseline Values	3-Month Follow-Up	Change	Baseline Values	3-Month Follow-Up	Change	
Lipid profile							
Cholesterol (mmol/L)	4.9 ± 0.9	4.8 ± 0.9	−0.1 ± 0.8	4.9 ± 0.9	4.9 ± 1.2	−0.1 ± 0.7	0.79
TAG (mmol/L)	2.2 ± 1.0	1.6 ± 0.7	−0.6 ± 0.9 **	2.3 ± 2.0	1.6 ± 0.7	−0.7 ± 1.6	0.86
LDL (mmol/L)	2.9 ± 0.8	2.9 ± 0.9	+0.1 ± 0.7	2.9 ± 0.8	3.1 ± 1.0	+0.2 ± 0.6	0.44
HDL (mmol/L)	1.1 ± 0.2	1.2 ± 0.3	+0.1 ± 0.1 *	1.2 ± 0.3	1.2 ± 0.3	0.0 ± 0.2	0.15
Liver enzymes							
ALT (µkat/L)	0.8 ± 0.7	0.7 ± 0.5	−0.1 ± 0.4	0.8 ± 0.3	0.7 ± 0.3	−0.1 ± 0.2 *	0.57
AST (µkat/L)	0.6 ± 0.4	0.4 ± 0.2	−0.1 ± 0.4 *	0.6 ± 0.3	0.5 ± 0.4	−0.1 ± 0.2	0.31
GGT (µkat/L)	0.7 ± 0.4	0.7 ± 0.4	−0.1 ± 0.2	0.7 ± 1.0	0.7 ± 0.9	−0.1 ± 0.2	0.97

All data are presented as mean ± SD; * *p* < 0.05; ** *p* < 0.01.

**Figure A1:**
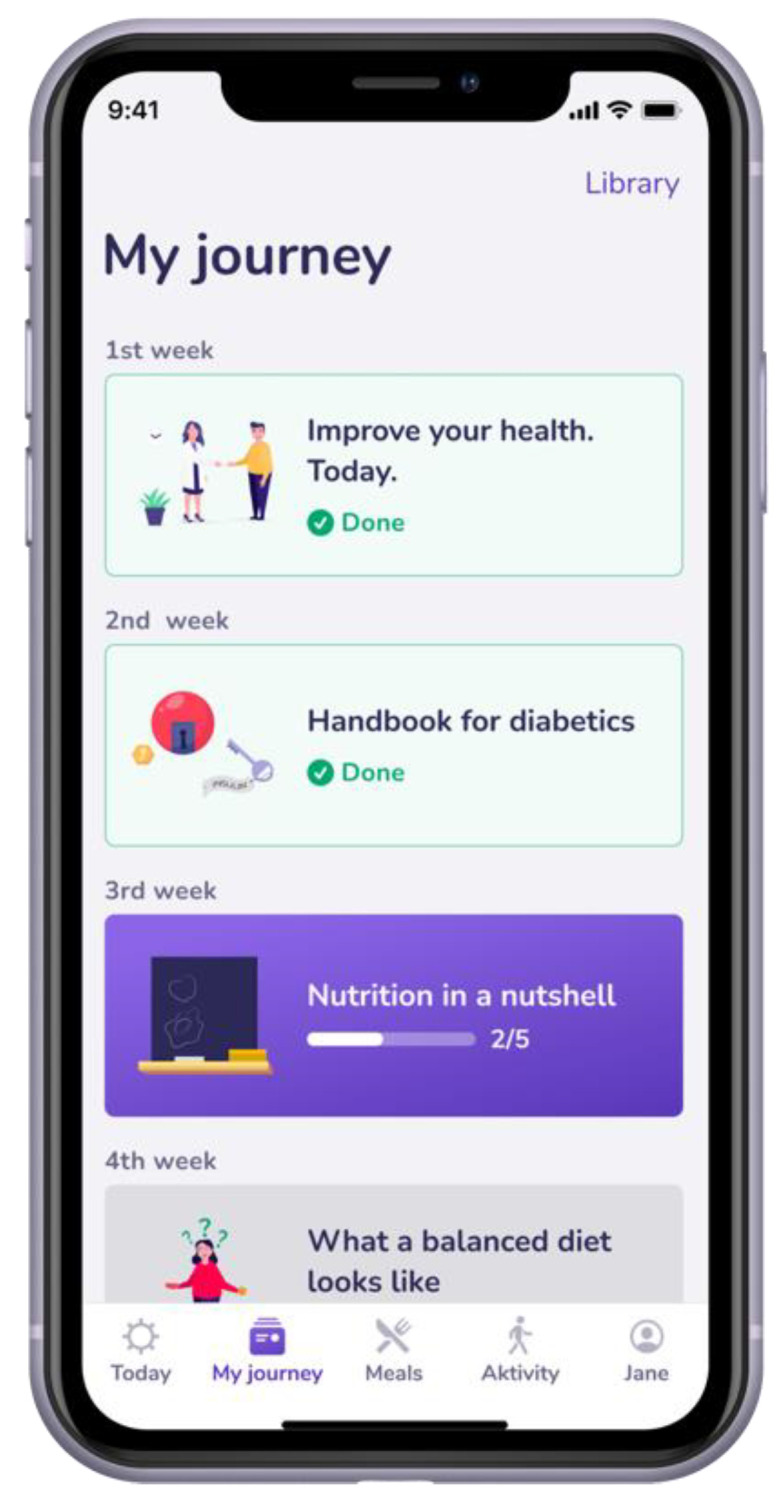
Interactive education in the app.

**Figure A2:**
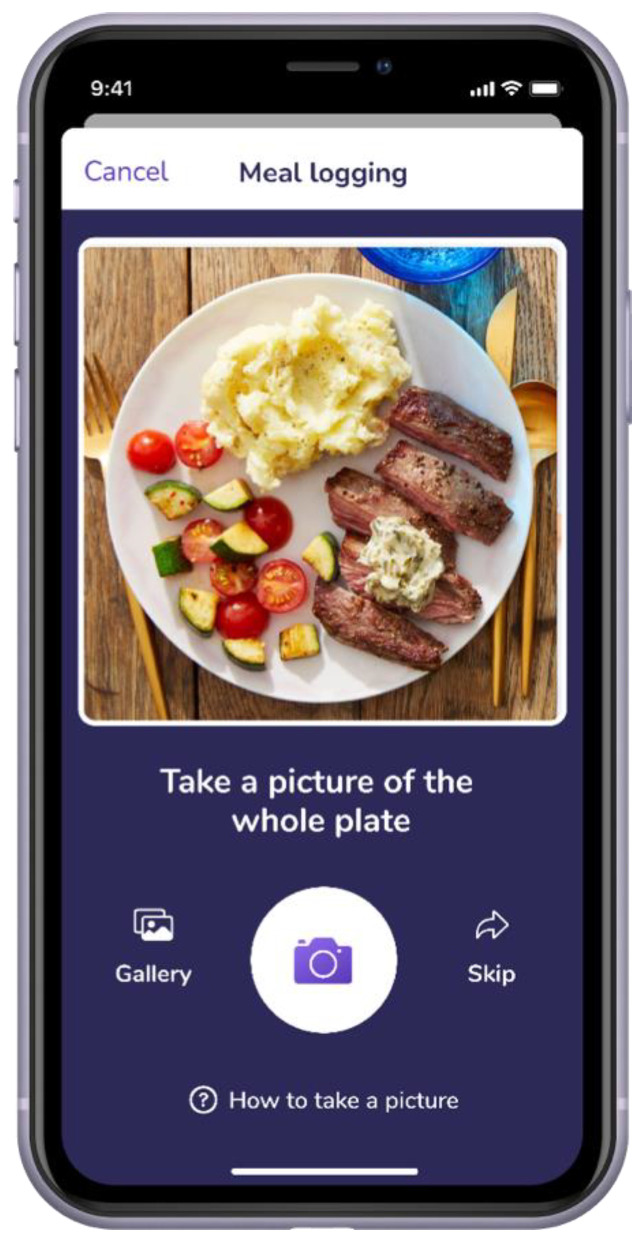
In-app meal-logging function.

**Figure A3:**
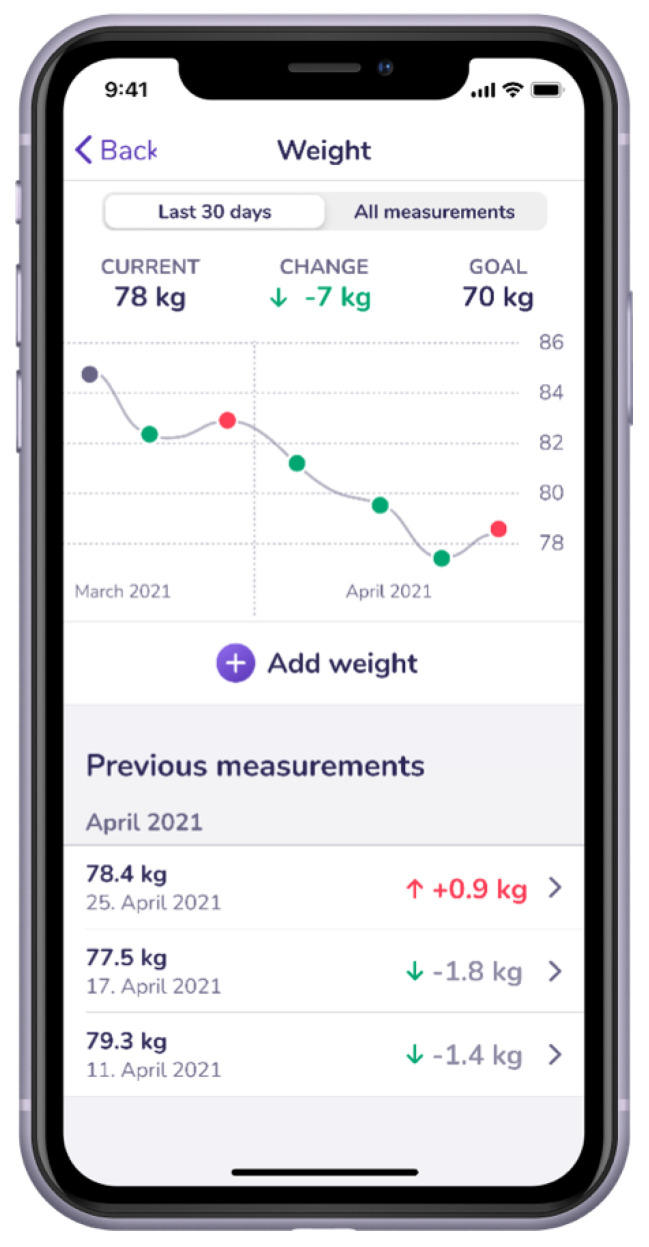
Tracking of weight change.

**Figure A4:**
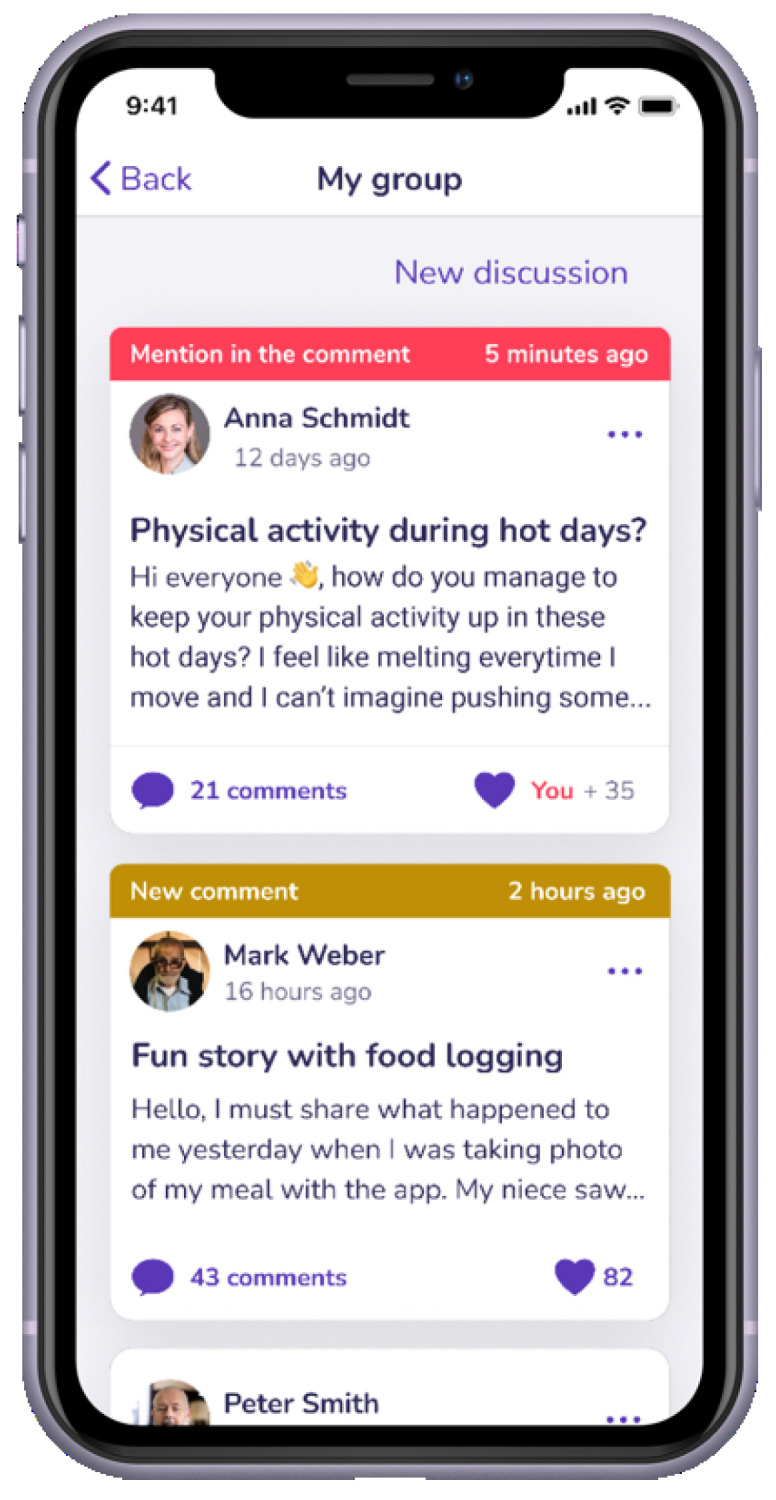
In-app peer support group.

## Data Availability

The data presented in this study are available to researchers who submit a methodologically sound proposal to the corresponding author, K.M. (katarina.moravcova@fnol.cz). To gain access to the data, proposers will be required to sign a data access agreement.
